# Shwachman-Diamond syndrome: a complex case demonstrating the potential for misdiagnosis as asphyxiating thoracic dystrophy (Jeune syndrome)

**DOI:** 10.1186/1471-2431-12-48

**Published:** 2012-05-03

**Authors:** Steven J Keogh, Shane McKee, Sarah F Smithson, David Grier, Colin G Steward

**Affiliations:** 1 Department of Paediatric Haematology, Oncology & BMT, Royal Hospital for Children, University Hospitals Bristol NHS Foundation Trust, Upper Maudlin St, Bristol, BS2 8BJ, UK; 2 Northern Ireland Regional Genetics Service, Belfast City Hospital, Lisburn Rd, Belfast, Northern Ireland, BT9 7AB, UK; 3 Department of Clinical Genetics, St Michael’s Hospital, Southwell Street, Bristol, BS2 8EG, UK; 4 Department of Paediatric Radiology, Royal Hospital for Children, University Hospitals Bristol NHS Foundation Trust, Upper Maudlin St, Bristol, BS2 8BJ, UK; 5 Department of Cellular & Molecular Medicine, School of Medical Sciences, University Walk, Bristol, BS8 1TD, UK

**Keywords:** Shwachman-Diamond syndrome, Asphyxiating thoracic dystrophy, Jeune syndrome, Differential diagnosis, Haematopoietic stem cell transplantation, Isochromosome 7q, Pancreatic insufficiency, Neonatal respiratory distress

## Abstract

****Background**:**

The differential diagnosis of a neonate or fetus presenting with a bell-shaped or long narrow thorax includes a wide range of bony dysplasia syndromes. Where this is accompanied by respiratory distress, asphyxiating thoracic dystrophy (ATD, Jeune syndrome) is an important potential diagnosis. Shwachman-Diamond syndrome (SDS) is widely recognised as a cause of exocrine pancreatic dysfunction, short stature and bone marrow failure. It is not so well appreciated that rib and/or thoracic cage abnormalities occur in 30–50% of patients and that, in severe cases, these abnormalities may lead to thoracic dystrophy and respiratory failure in the newborn. There are, however, at least three previous case reports of children who were initially diagnosed with ATD who were subsequently shown to have SDS.

****Case presentation**:**

This report details the case history of a patient misdiagnosed as having ATD as a neonate following the neonatal asphyxial death of her brother. She subsequently developed progressive pancytopenia but was only diagnosed with SDS at 11 years of age after referral for haematopoietic stem cell transplantation for bone marrow failure accompanied by trilineage dysplasia and clonal cytogenetic abnormalities on bone marrow examination. Subsequent testing revealed the presence of fat globules in stools, reduced faecal chymotrypsin, fat-soluble vitamin deficiency, metaphyseal dysplasia on skeletal survey and heterozygous mutations of the SBDS gene.

****Conclusion**:**

This report highlights the potential for diagnostic confusion between ATD and SDS. It is important to include SDS in the differential diagnosis of newborns with thoracic dystrophy and to seek expert clinical and radiological assessment of such children.

## Background

The presentation of a bell-shaped or long narrow thorax is often associated with short limbs and sometimes polydactyly. The differential diagnosis for this presentation is wide and includes asphyxiating thoracic dystrophy (ATD, Jeune syndrome, OMIM 208500), Ellis-van Creveld syndrome, short rib-polydactyly syndrome (types I-IV), thoracolaryngopelvic dysplasia (Barnes syndrome) and Shwachman-Diamond syndrome (SDS, OMIM 260400). In this article we have concentrated solely on the risk of diagnostic confusion between ATD and SDS.

ATD was first described by Jeune et al. in a pair of siblings in 1955 [1]. It is an autosomal recessive disorder characterised by chondrodysplasia and multiple organ involvement [2] with an incidence of one case per 100,000 to 130,000 live births [3]. Diagnosis in the prenatal or neonatal period is based on clinical and radiological findings. Two causative genes have recently been identified, IFT80 at 3q24‐q26 [4] and DYNC2H1 at 11q14.3‐q23.1 [5], and several loci have been implicated in those lacking mutations of these genes.

Skeletal changes in ATD include a narrow chest and disproportionately short limbs. All patients have small chests but the clinical phenotype varies widely, with some children dying in the neonatal period from asphyxia but others having milder respiratory symptoms. Patients are increasingly surviving the neonatal period due to better intensive care therapies, which in turn allow them to reach an age where there is decreased reliance on chest wall mechanics for ventilation [2,6]. As a result, while patients in the past rarely survived childhood, some are now living to adulthood where they often display chronic renal failure and other manifestations of the syndrome. Extraosseous manifestations particularly affect the kidney, liver and pancreas. Hepatic fibrosis and pancreatic cystic disease and fibrosis have all been well described [7].

SDS is an autosomal recessive disorder characterised primarily by exocrine pancreatic dysfunction, short stature, metaphyseal dysplasia and bone marrow failure [8]. The gene responsible, SBDS at 7q11, was identified in the last decade [9,10] and mutations are identifiable in over 75% of patients with the syndrome. Haematological involvement may cause isolated cytopenias or pancytopenia, and there is a propensity to develop myelodysplasia (MDS) or acute myeloid leukaemia [11]. The commonest cytopenia at diagnosis is neutropenia. This is seen in 88–100% of SDS patients from neonatal life onwards, although neutrophil counts can vary from severely low to normal [8]. Imaging studies show pancreatic lipomatosis [12].

Published case series in SDS [13-15] describe metaphyseal chondroplasia in 40‐80% of patients and short stature in more than 50%. Other skeletal abnormalities may include clinodactyly, kyphosis, scoliosis, coxa vara, vertebral collapse, slipped femoral epiphysis, supernumerary metatarsals, genu and cubitus valgus, pes cavus and osteopenia. Rib and/or thoracic cage abnormalities occur in 30‐50% of patients. In severe cases, these abnormalities may lead to thoracic dystrophy and respiratory failure in the newborn period [8].

The differential diagnosis of a neonate or fetus presenting with a bell-shaped or long narrow thorax includes a wide range of bony dysplasia syndromes. Where this is accompanied by respiratory distress, ATD is an important potential diagnosis. SDS must, however, be considered in this differential and be excluded by appropriate investigation. There are historical references to at least three cases of children initially diagnosed with ATD, who were subsequently shown to have SDS [16-19]. This report details the case history of another patient misdiagnosed as having ATD as a neonate following the neonatal asphyxial death of her brother. She was then diagnosed with SDS at 11 years of age after referral for haematopoietic stem cell transplantation for bone marrow failure. It highlights the potential for diagnostic confusion between ATD and SDS in neonates with thoracic dystrophy and the need for expert clinical and radiological assessment of such children.

## Case presentation

**Figure 1:**
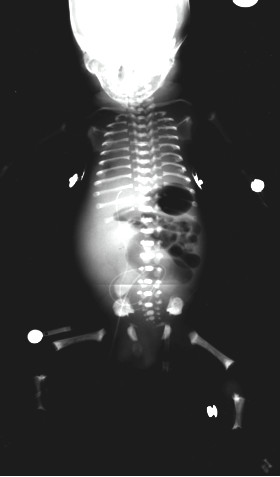
**Babygram of the affected male sibling with severe respiratory distress.** There is mild platyspondyly and generalised shortening of ribs and long bones. The metaphyses of the long bones are slightly irregular and there is some metaphyseal sclerosis.

An 11-year-old girl (UPN1) was referred for evaluation for matched sibling bone marrow transplantation (BMT) for treatment of MDS. She was the youngest of three children born to unrelated parents. The first male born to her parents was small for gestational age, weighing 1.87 kg at term. He had severe chest deformity (babygram shown in Figure 1) and died of respiratory distress at four hours of age. A diagnosis of probable ATD was made although no genetic testing could be performed, as causative genes were unknown at that time. The second was a well, unaffected male (who subsequently became the bone marrow donor for UPN1).

UPN1 was born at 40 + 6 weeks gestation by a normal vaginal delivery. Her Apgar scores were 6 at one minute and 8 at 5 minutes and she required suction and oxygen by mask. Her birth weight was 2.59 kg, length 47 cm and head circumference 33 cm (all approximately on the 3^rd^ percentile). From 12 to 36 hours of postnatal life she developed tachypnoea, reaching a maximum of 120 breaths per minute. There was no cyanosis. On examination she had an obvious narrow chest, poor chest expansion, hepatomegaly of 2 cm and significant tachypnoea but no other obvious abnormalities. Chest auscultation was clear and cardiac examination was normal. A chest radiograph (not available) showed a narrow chest with no evidence of cardiac disease and the lung fields were consistent with transient tachypnoea of the neonate. UPN1 was admitted to the neonatal unit and was discharged 48-hour later with mild tachypnoea only. A geneticist had reviewed her and a diagnosis of probable ATD was reached. At this time her haemoglobin and platelet counts were normal and her total white cell count was 5.2 x 10^9^/l (no differential performed). Shortly afterwards her respiratory symptoms resolved completely.

Through the first two years of life head circumference stayed at the third percentile and her length percentile dropped. By two years she was 10 cm below the 3^rd^ percentile for height and 2 kg below the 3^rd^ percentile for weight. At 22 months of age she was admitted with sepsis with a short history of vomiting, pyrexia and a rapidly progressive purpuric rash. Meningococcal septicaemia was the presumed diagnosis and she was fully treated for this but meningococcus was not cultured and coliforms were grown subsequently from urine. Haemoglobin on admission was 4.6 g/dl, white cell count was 7.8 x 10^9^/l (neutrophils 2.34 x 10^9^/l) and the platelet count was 18 x 10^9^/l. These abnormalities were presumed to be secondary to severe sepsis and she made a rapid and full response to intravenous antibiotics. Ten days later her neutrophils had dropped to 1.14 x 10^9^/l but platelets had recovered to 53 x 10^9^/l. There is no documented full blood examination until 5 years of age when she developed haemolytic uraemic syndrome (HUS) and required several weeks of dialysis; this was followed by intermittent, self-limiting episodes of haematuria with mild renal impairment. At the time of admission with HUS she was noted to have learning difficulties and short stature. Renal imaging at the age of 7 years with ultrasound and DMSA showed no renal scarring, malformation, dilatation or reflux. At this time and for the following 4 years her blood counts were normal (including neutrophils) apart from a platelet count ranging from 43‐161 x 10^9^/l but mostly in the range 50–100 x 10^9^/l.

Between the ages of 9 and 10 years UPN1 progressed to being consistently pancytopenic (haemoglobin 6.8 g/dl, white cell count 1.8 x 10^9^/l, neutrophils 0.47 x 10^9^/l, lymphocytes 1.09 x 10^9^/l and platelets of 33 x 10^9^/l). She had no evidence of hepatosplenomegaly or lymphadenopathy. A bone marrow aspirate and trephine showed normal cellularity with trilineage dysplasia and no fibrosis. There was no excess of blasts initially, although repeat examination after referral showed 10% blasts and the marrow had become hypocellular. Cytogenetics revealed a deletion of 7p in a clone on the initial bone marrow aspirate but serial testing eventually revealed three different clones, one with an isochromosome of 7q, an abnormality frequently described in SDS and rarely seen outside this syndrome [8]. Lymphocytes showed no increase in chromosome breakage compared to control in response to mitomycin C challenge, excluding Fanconi anaemia.

UPN1 became red cell and platelet transfusion dependent in the year leading up to transplant referral. During this time she suffered epiglottitis requiring intensive care, a prolonged episode of fever with a perianal abscess and periorbital cellulitis of one eye. Perianal soreness and infection became a recurring theme.

**Figure 2:**
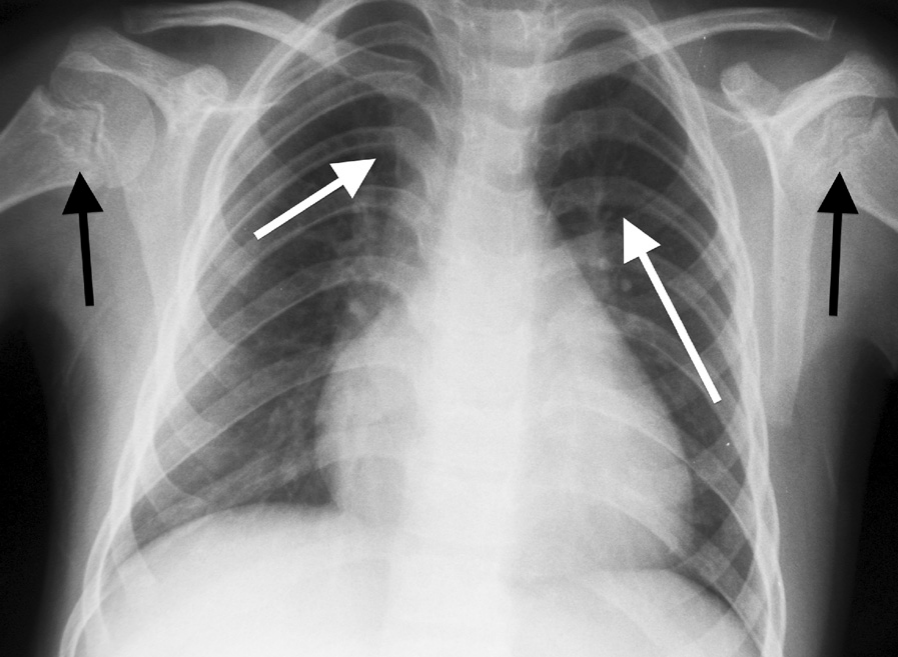
**Chest radiograph of UPN1 at referral for BMT aged 11 years.** There is mild metaphyseal sclerosis and irregularity of the proximal humeri (black arrows). The posterior ribs are bowed (white arrows) and there is the impression of cardiomegaly due to the slightly narrowed thorax.

**Figure 3:**
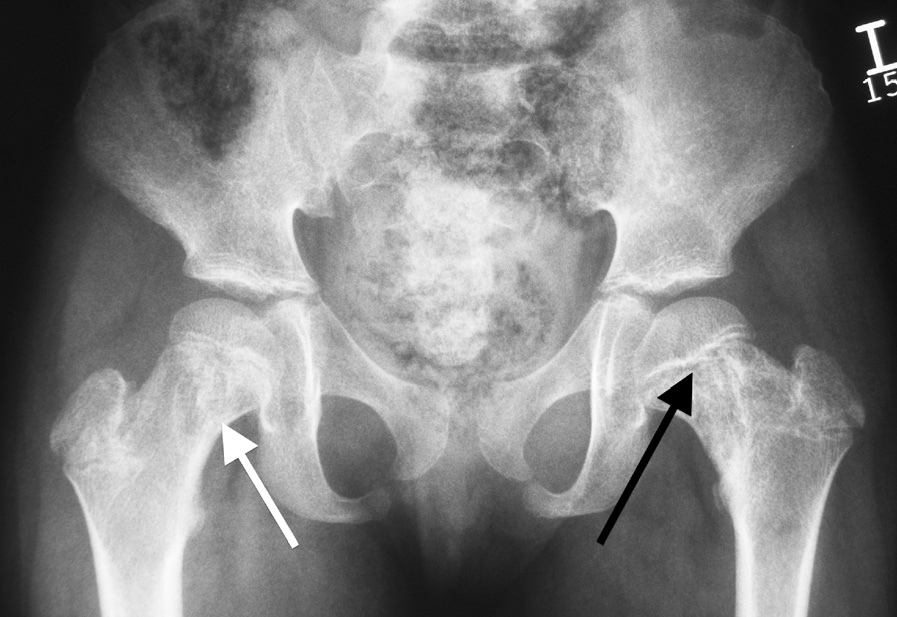
**Pelvis radiograph of UPN1 at referral for BMT.** There is prominent sclerosis and lucency in both proximal femoral metaphyses (black arrow) and femoral necks (white arrow). These changes extend into the subtrochanteric regions. The acetabular roofs are horizontal.

**Figure 4:**
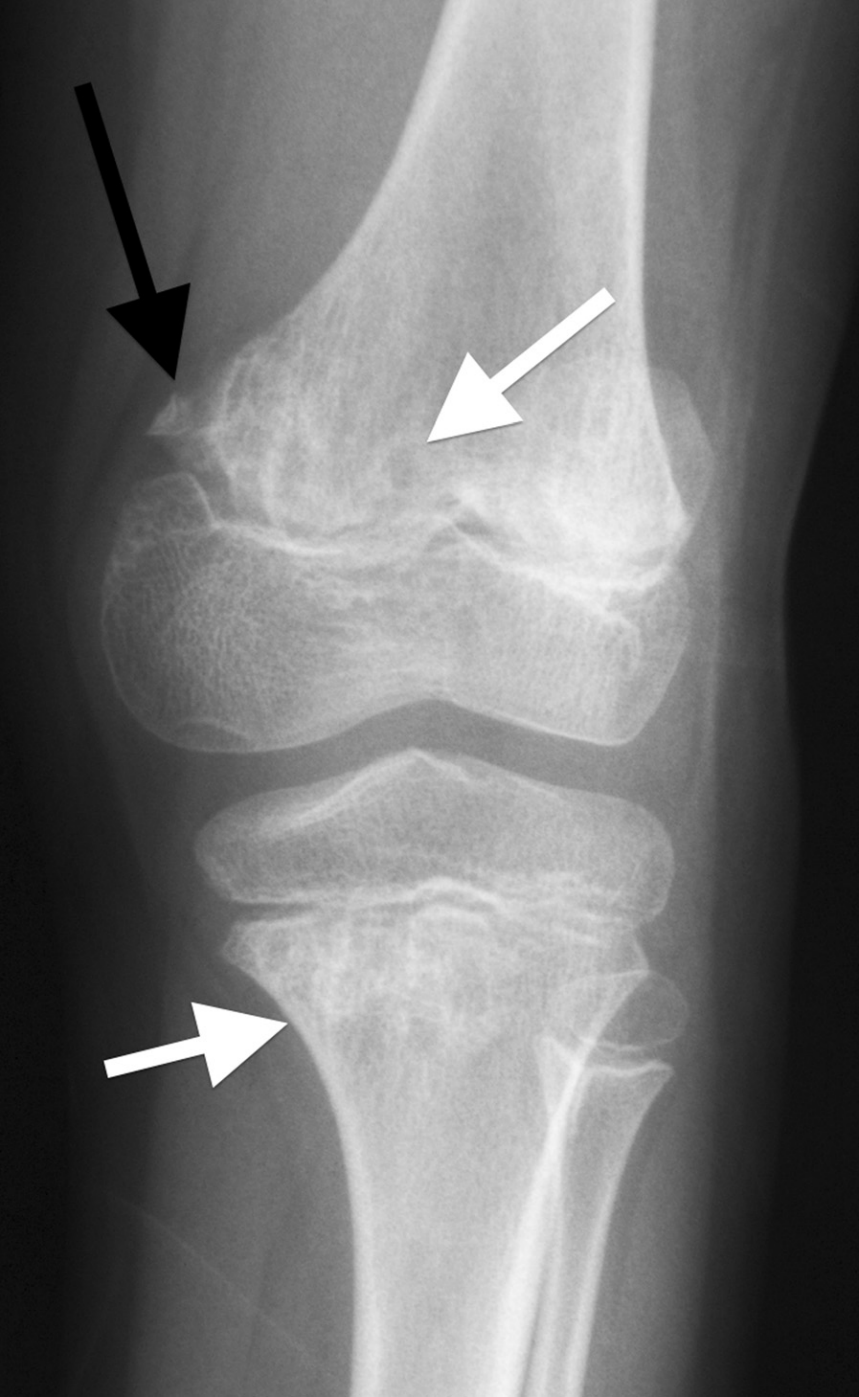
**Knee radiograph of UPN1.** There is sclerosis and lucency in the distal femoral and proximal tibial metaphyses (white arrows) with pseudofragmentation of the medial part of the distal femoral metaphysis (black arrow).

Specific questioning to exclude well-recognised causes of bone marrow failure revealed that UPN1 had passed 1‐2 stools per day throughout her life, which were yellow, offensive and difficult to flush. Microbiological examination had been consistently negative. Subsequent examination revealed the presence of fat globules and a reduced faecal chymotrypsin (0.7U/g, normal range 6‐99). Fat-soluble vitamin levels were reduced (vitamin A 0.6 μmol/l [reference range 1.1‐3.5] and vitamin E 9.7 μmol/l [reference range 10.2‐39.0]). UPN1 was growing but was still small for age; at eleven years and two months she weighed 22.9 kg (5 kg below 3^rd^ percentile) and was 113 cm tall (approximately 20 cm below the 3^rd^ percentile). Skeletal survey (See Figures 2, 3 and 4) revealed abnormalities in the metaphyseal regions, most prominent in the upper femoral neck and in the lower femoral and upper tibial metaphyses. These showed areas of lucency and sclerosis and irregularity. Coxa valga was present on the right and some broadening and coxa vara on the left. The ribs were a little shortened.

The radiographic changes on skeletal survey in combination with the evidence of pancreatic insufficiency, confirmed a clinical diagnosis of SDS as a cause of her chondroplasia and bone marrow failure. A DNA sample from UPN1 was later tested for conversion mutations of the SBDS gene. The restriction endonuclease digest assay confirmed mutations c.183_184TA>CT and c.258+2T>C. These account for 74% of SBDS mutations [9]. Testing of parental DNA also confirmed that one mutated allele was inherited from each parent.

UPN1 had significant learning difficulties and was in a special school. MRI brain was unremarkable. She was commenced on fat-soluble vitamins and pancreatic enzyme replacement with rapid improvement in stool frequency and consistency. An MRI scan of the abdomen showed that the pancreas was bulky and infiltrated with fat. At referral she was receiving packed cell transfusions every three weeks and platelets approximately weekly, but blood counts improved rapidly following pancreatic and vitamin supplementation. During the following 16 months, she required only one further packed cell transfusion and became platelet independent, although she did require G-CSF in order to increase her neutrophil count. She had minimal infectious complications. Due to this improvement, plans for transplantation were postponed.

**Figure 5:**
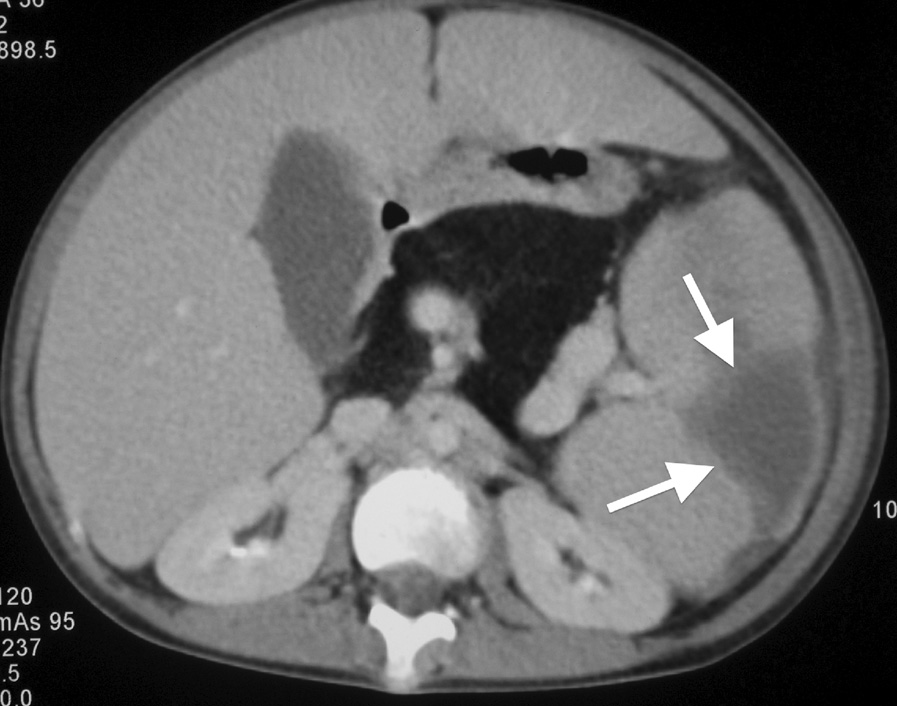
**Contrast enhanced computed tomography (CT) of abdomen.** The spleen is enlarged and contains a well-defined wedge shaped peripheral zone of low attenuation which does not enhance consistent with infarction (arrows). There is enlargement and tortuosity of the splenic vein. The pancreas is enlarged and of uniform low attenuation consistent with fatty replacement.

**Figure 6:**
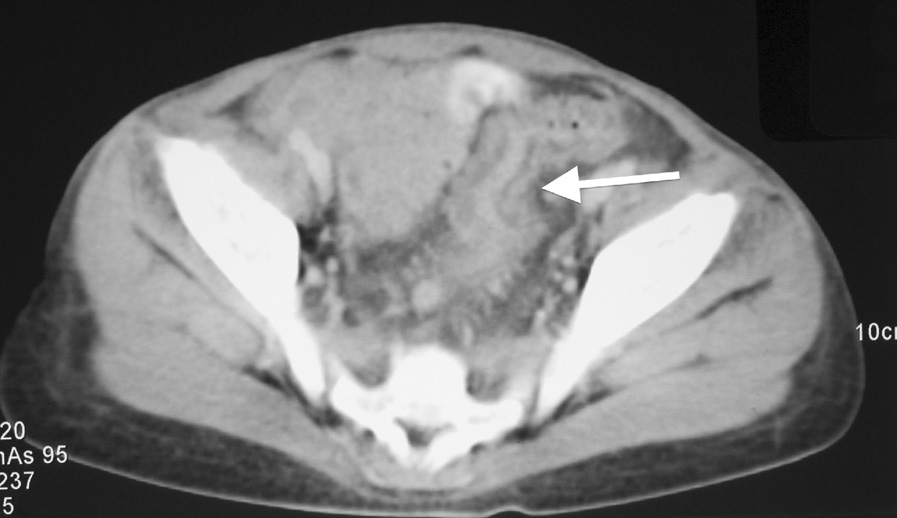
**Contrast enhanced computed tomography (CT) of abdomen.** There is wall thickening of the sigmoid colon with stranding of adjacent mesenteric fat (arrow). The appearances are of colitis.

When UPN1 was 12.5 years old, her blood count fell further, she developed pyrexia of unknown origin (PUO) lasting four weeks and was admitted for intravenous antibiotics. During this time her spleen enlarged to 14 cm below the costal margin, causing significant abdominal discomfort. The PUO settled and she was discharged but returned 4 months later with recurrence of fever and further progression of splenomegaly. Extensive imaging was performed during these two admissions. Chest radiographs and CT scans initially showed no focus of infection. There was hepatosplenomegaly and, of note, the pancreas was enlarged with markedly reduced attenuation consistent with being almost completely replaced by fat (See Figure 5). There was also mural thickening of the sigmoid colon and rectum, suggesting neutropenic colitis/proctitis as the cause of PUO (See Figure 6). Lung function studies revealed both FVC and FEV1 in the low normal range.

Progressive splenomegaly was thought to be related to progression of MDS and chronic G-CSF exposure as imaging showed no evidence of portal hypertension. UPN1 continued to have fevers resistant to intravenous antibiotics with no evidence of invasive fungal disease. Splenectomy was performed due to concerns that splenic sequestration was contributing to decreasing blood counts. Pathology revealed a large spleen (737 grams compared with mean weight for age being 89 grams) with several areas of infarction. The white pulp was atrophic and the red pulp congested. Microscopy showed areas of infarction with diffuse expansion of the red pulp, resulting from extensive infiltration of left shifted myeloid cells and clusters of atypical blasts. These features were thought to be attributable to MDS. Rectal biopsy was also performed, revealing features consistent with neutropenic colitis.

Persistent neutropenia, abdominal pain and recurrent fevers necessitated matched sibling BMT after conditioning therapy comprising fludarabine (125 mg/m^2^), melphalan (140 mg/m^2^) and alemtuzumab (0.9 mg/kg). This procedure was complicated by primary graft rejection, necessitating re-transplantation following administration of fludarabine (100 mg/m^2^), high dose methylprednisolone (10 mg/kg twice daily for four doses, then 2 mg/kg twice daily for four doses followed by slow weaning) and OKT3 (0.2 mg/kg for eight doses). This was followed by rapid and uncomplicated donor engraftment. Mixed T-cell chimerism led to donor lymphocyte infusions to reduce the risk of relapse of MDS. UPN1 is now over 5 years post-transplant and is well with normal blood counts. Whole blood and T-cell chimerism levels are 98% and 89% donor respectively. Hepatomegaly resolved rapidly following successful engraftment.

## Conclusions

SDS poses a number of diagnostic challenges when it presents with severe neonatal respiratory distress. Firstly, although previously described [8], this presentation is rare. Secondly and very importantly, many of the cardinal symptoms such as steatorrhoea, failure to thrive and neutropenia may not be apparent soon after birth, or not be recognised unless the information is specifically sought. As many SDS patients have improvement in pancreatic function over time and the neutropenia fluctuates, the diagnosis may become more difficult to establish as the child develops. Thirdly, the characteristic chondrodysplasia often seen in this condition may well not become apparent until the second or third year of life. Thus, a child with neonatal respiratory distress consequent on severe thoracic dystrophy is much more likely to be diagnosed with a primary skeletal dysplasia, such as ATD, than with SDS. SDS is also largely managed by gastroenterologists and haematologists, rather than those who would likely see a neonate in respiratory distress.

There is a further serious source of confusion, as pancreatic insufficiency is said to occur in both diseases. It is a major component of SDS, the pancreatic stimulation test being abnormal in 100% of cases in the three major published series of SDS [13-15], and occurs far earlier than in ATD. The association of pancreatic insufficiency and ATD is widely cited in articles and textbooks, as well as on various websites and online resources. However, the excellent review of ATD by de Vries et al. [2] identifies only two articles which detail this link.

The first, by Karjoo et al. in 1973 [17] described a neonatal sibling pair who were thought to have ATD and had pancreatic insufficiency. However, Danks et al. from 1976 [18], reported two sets of siblings with metaphyseal chondrodysplasia, neutropenia and pancreatic insufficiency who presented in the neonatal period with respiratory distress. One set were the same children described previously by Karjoo et al. as having ATD. It seems almost certain that these four children all suffered from SDS, although testing for the SBDS gene was not available at that time. Unfortunately, modern literature searches do not pick up on the fact that one of the sibling pairs described by Karjoo was later realised to have SDS. Therefore, reviews of ATD continue to associate pancreatic insufficiency with ATD.

The second paper mentioned by de Vries, written by Turkel et al. [20] from 1985 (again prior to the capacity to genetically test for SDS) reported the necropsy findings of seven newborn infants with presumed ATD. In the discussion it states, “pancreatic exocrine insufficiency is a rare complication seen in older patients with Jeune ATD” and references only one paper from 1978, which we are unable to obtain. We believe that it is reasonable to assume that pancreatic insufficiency is therefore not a feature of ATD in childhood. Although pathological examination reveals cystic changes and fibrosis on necropsy, it is unlikely that these become clinically significant until late in life (if ever). Thus, if a child with thoracic dystrophy has evidence of pancreatic insufficiency, we suggest that pancreatic imaging, pancreatic function testing and SBDS genotyping should be performed.

In the case that we have reported here, the initial diagnosis was made in an index male sibling whose thoracic dystrophy was so severe that he died within the first 24 hours of life. Clearly, there would have been nothing on which to base a potential diagnosis of SDS at that stage. When his sister was also born with thoracic dystrophy, it was natural for the clinical team concerned to conclude that this was a second case of the same disease. A full blood examination on day two of life was normal although no white cell differential is available from this time. Blood count abnormalities were evident at 22 months of age but only in the context of severe sepsis. Neutropenia was not a prominent and consistent feature until 9 years of age. The subsequent (and still unexplained) development of haemolytic uremic syndrome was thought to be due to ATD, since renal lesions have been described. This distracted from critical analysis of other features, such as evolving cytopenia and extra-thoracic infections, that were inconsistent with the classical picture of ATD.

Since 2007 several causative genes for forms of ATD have been described, although in most cases no causative gene is yet identified. Tests which may contribute to the exclusion of SDS include measurement of faecal fat, serum trypsinogen levels, imaging looking for pancreatic lipomatosis (See Figure 5) and scrutinising the blood count for neutropenia (defined as the neutrophil count of less than 1 x 10^9^/l in a child of less than one year of age) on more than one occasion. Expert review of radiology should be able to distinguish the two conditions in infancy (personal communication, Professor Christine Hall). If clear diagnosis is not possible in the neonatal period we would advise referral to a specialist skeletal dysplasia service. Unless definitive genetic testing is done and confirms a diagnosis, we also suggest keeping an open mind, as diagnostic certainty can be gained with time.

This case raises a second broader concern, which is that, once associations have been described, they are easily perpetuated through internet sites and the medical literature, even if they are subsequently disproven or discredited. For example, at the time that we were first investigating this patient one specialist website on Jeune syndrome gave a detailed account of a child believed to have that disease who was neutropenic and on pancreatic supplements. This patient was subsequently reclassified as having SDS.

Accurate diagnosis of these children has major implications for management, prognosis, and counselling of the affected individuals and their families. We, therefore, advise expert clinical and radiological review of patients with neonatal thoracic dystrophy. Where a genetic diagnosis of ATD is not confirmed, pancreatic lipomatosis and evolving neutropenia should be excluded.

## Consent

Written informed consent for publication of this case report and imaging for both UPN1 and her sibling was obtained from the parents. A copy of the written consent is available for review by the Editor-in-Chief of this journal.

## Abbreviations

ATD, asphyxiating thoracic dystrophy; BMT, bone marrow transplant; CT, computed tomography; DNA, deoxyribonucleic acid; DMSA, 99mTc-dimercaptosuccinic acid renal imaging; FEV1, forced expiratory time in 1 second; FVC, forced vital capacity; G-CSF, granulocyte colony stimulating factor; HUS, haemolytic uraemic syndrome; MDS, myelodysplastic syndrome; MRI, magnetic resonance imaging; OKT3, brand name for muromonab-CD3; PUO, pyrexia of unknown origin; SDS, Shwachman-Diamond syndrome.

## Competing interests

The authors declare they have no competing interests.

## Authors’ contributions

SJK and CGS conceived the paper. SJK performed literature searches, and drafted the manuscript. CGS assisted in writing the manuscript and edited. DG edited the manuscript, provided the imaging and assisted with radiology opinion and image legends. SFS edited the manuscript and gave expert opinion on bony dysplasia and genetics. SM edited the manuscript, gained parental consent and organized genetic testing. All authors read and approved the final manuscript.

## Authors’ information

SJK is a Consultant in Bone Marrow Transplantation at the Children’s Hospital at Westmead, Australia, with interest in bone marrow failure syndromes. SM is a Consultant in Clinical Genetics at the Northern Ireland Regional Genetics Service in Belfast with a special interest in cancer predisposition syndromes. SFS is Consultant in Clinical Genetics at St Michael’s Hospital, Bristol with a special interest in dysmorphology and skeletal dysplasias. DG is Consultant in Paediatric Radiology at Bristol Royal Hospital for Children. CGS is a Consultant in haematopoietic stem cell transplantation at Bristol Royal Hospital for Children specialising in transplantation of aplastic anaemia, bone marrow failure syndromes and other genetic diseases with a specific interest in factors leading to misdiagnosis of rare genetic diseases.

## Pre-publication history

The pre-publication history for this paper can be accessed here:

http://www.biomedcentral.com/1471-2431/12/48/prepub
